# Relationship between history of hormonal contraceptive use and anaemia status among women in sub-Saharan Africa: A large population-based study

**DOI:** 10.1371/journal.pone.0286392

**Published:** 2023-06-14

**Authors:** Richard Gyan Aboagye, Joshua Okyere, Abdul-Aziz Seidu, Bright Opoku Ahinkorah, Eugene Budu, Sanni Yaya

**Affiliations:** 1 Department of Family and Community Health, Fred N. Binka School of Public Health, University of Health and Allied Sciences, Hohoe, Ghana; 2 Department of Population and Health, University of Cape Coast, Cape Coast, Ghana; 3 Department of Nursing, College of Health Sciences, Kwame Nkrumah University of Science and Technology, Kumasi, Ghana; 4 REMS Consult Limited, Takoradi, Western Region, Ghana; 5 College of Public Health, Medical and Veterinary Sciences, James Cook University, Douglas, Queensland, Australia; 6 School of Public Health, Faculty of Health, University of Technology Sydney, Sydney, Australia; 7 Research Unit, Korle Bu Teaching Hospital, Accra, Ghana; 8 School of International Development and Global Studies, University of Ottawa, Ottawa, Canada; 9 The George Institute for Global Health, Imperial College London, London, United Kingdom; Kwame Nkrumah University of Science and Technology College of Health Sciences, GHANA

## Abstract

**Background:**

Anaemia among women has been reported to be a significant contributor to hemorrhage, exacerbated risk of stillbirths, miscarriages, and maternal mortalities. Hence, understanding the factors associated with anaemia is imperative to develop preventive strategies. We examined the association between history of hormonal contraceptive use and risk of anaemia among women in sub-Saharan Africa.

**Methods:**

We analyzed data from the recent Demographic and Health Surveys (DHS) of sixteen countries in sub-Saharan Africa. Countries with recent DHSs conducted from 2015 to 2020 were included in the study. A total of 88,474 women of reproductive age were included. We used percentages to summarize the prevalence of hormonal contraceptives and anaemia among women of reproductive age. We used multilevel binary logistic regression analysis to examine the association between hormonal contraceptives and anaemia. We presented the results using crude odds ratio (cOR) and adjusted odds ratios (aOR), with their respective 95 percent confidence intervals (95% CIs).

**Results:**

On the average, 16.2% of women are using hormonal contraceptives and this ranged from 7.2% in Burundi to 37.7% in Zimbabwe. The pooled prevalence of anaemia was 41%, ranging from 13.5% in Rwanda to 58.0% in Benin. Women who used hormonal contraceptives were less likely to be anaemic compared to those who were not using hormonal contraceptives (aOR = 0.56; 95%CI = 0.53, 0.59). At the country-level, hormonal contraceptive use was associated with a reduced likelihood of anaemia in 14 countries, except for Cameroon and Guinea.

**Conclusion:**

The study underscores the importance of promoting the use of hormonal contraceptives in communities and regions that have a high burden of anaemia among women. Specifically, health promotion interventions aimed at promoting the use of hormonal contraceptives among women must be tailored to meet the needs of adolescents, multiparous women, those in the poorest wealth index, and women in union as these sub-populations were at significantly higher risk of anaemia in sub-Saharan Africa.

## Background

Since the ratification of the Programme of Action during the 1994 International Conference on Population and Development held in Cairo, Egypt, there has been a high priority on the sexual and reproductive health (SRH) issues of women at the global level [1]. One of such SRH priorities is the use of hormonal contraceptives. Hormonal contraceptive is a type of contraceptive that administers “exogenous hormones that affect the endocrine regulation of the female reproductive system and may inhibit ovulation” [2]. The commonest form of hormonal contraceptives is oral contraceptives [2]. However, other forms of hormonal contraceptives include implants, skin patches, hormone-releasing contraceptive coils, and intravaginal rings [3]. Generally, Hormonal contraceptives may either contain only a progestin component, or a combination of both estrogenic and progestin components [4].

A 2019 report by the United Nations revealed that globally, 50% of the 1.1 billion women who used family planning were users of hormonal contraceptives [5]. Evidence shows that correct and consistence use of hormonal contraceptives are one of the most effective, safe, and reversible forms of contraception [6]. Nevertheless, there is growing concern about the association between the use of hormonal contraceptives and adverse health effects including anaemia [7, 8]. Thus, emphasizing an important need for evidence-based research to understand the association between the use of hormonal contraceptives and the risk of becoming anaemic.

Anaemia, which refers to a condition where the number of red blood cells in the body is insufficient to meet physiological needs, is a serious public health concern as nearly 1.2 billion people across the globe suffer from this ailment [9]. Women of reproductive age have a higher prevalence of anaemia (57%) than among any other sub-population; also, 40% of all pregnant women worldwide are anaemic [10, 11]. Anaemia among women has been reported to be a significant contributor to hemorrhage, decreased ability to tolerate blood loss, and exacerbated risk of stillbirths, miscarriages, and maternal mortalities [12, 13]. Hence, understanding the factors associated with anaemia is imperative to develop and implement preventive strategies.

Previous studies have shown that place of residence, level of education, wealth status, being overweight, ever terminating a pregnancy, and lack of autonomy to make healthcare decisions are associated with anaemia among women of reproductive age [14, 15]. Nevertheless, very few studies that have examined the association between the history of hormonal contraceptive use and the risk of anaemia. To the best of our knowledge after an extensive literature search, only one study conducted among Tanzanian women has investigated this association using a nationally representative data [7]. However, the findings of the Tanzanian study do not reflect the situation in sub-Saharan Africa (SSA). Hence, there is an inconclusive and significant literature gap at the sub regional level that needs to be filled. The present study, thus, examines the association between history of hormonal contraceptive use and the risk of anaemia among women using the most recent nationally representative data from 16 sub-Saharan African. The findings of this study are timely and necessary to inform targeted and tailored interventions to reduce anaemia within the sub-Saharan African sub. Policymakers and government across sub-Saharan African countries can use the recommendations from this study to inform policies and towards the achievement of the target 3.1 of the sustainable development goals (SDGs) which seeks to reduce maternal mortality to 70 per 100,000 live births [16].

## Methods

### Data source and study design

We analyzed data from the recent Demographic and Health Surveys (DHSs) of sixteen countries in SSA. Countries with recent DHS surveys conducted from 2015 to 2020 were included in the study. The countries consisted of Benin, Burundi, Cameroon, Ethiopia, Gambia, Guinea, Liberia, Mali, Malawi, Nigeria, Rwanda, Sierra Leone, Tanzania, Uganda, South Africa, and Zimbabwe. These countries had data on anaemia, history of hormonal contraceptives, and the covariates used in the study. DHS is a nationally representative survey conducted globally in over 90 low-and-middle-income countries [17, 18]. DHS aimed to improve demographic, health, and nutrition data collection, analysis, and distribution, as well as to make these data more useful for planning, policymaking, and programme management [17, 19]. In the DHS, a cross-sectional design was used. A two-stage cluster sampling technique was used to obtain the nationally representative data per place of residence and region. First, a predetermined number of enumeration areas (EAs) were selected using a probability proportional to the size of the list of EAs defined in the recent population census for a particular country. Later, a systematic sampling technique was adopted to select about 25 to 30 households per EAs for inclusion in the survey. At the household level, the entire household was enrolled in the survey. Detailed sampling methodology has been highlighted in the literature [19]. However, haemoglobin was only determined for a random sub-sample of women. In this study, 88,474 women of reproductive age with complete observations of variables of interest were included (Table 1). Pregnant women, women in the postpartum amenorrhea period, lactating mothers, and menopausal women were excluded. We draft this paper with reference to the Strengthening the Reporting of Observational Studies in Epidemiology (STROBE) guidelines [20]. The dataset is freely available to download at https://dhsprogram.com/data/available-datasets.cfm.

**Table 1:** Sample distribution and prevalence of anaemia and hormonal contraceptive use.

Country	Survey Year	Weighted sample	Weighted percentage	Anaemia	Hormonal contraceptives
1. Benin	2017–18	5,646	6.4	3273 (58.0)	465 (8.2)
2. Burundi	2016–17	6,002	6.8	2145 (35.7)	432 (7.2)
3. Cameroon	2018	5,146	5.8	2047 (39.8)	339 (6.6)
4. Ethiopia	2016	5,437	6.1	1182 (21.7)	1065 (19.6)
5. Gambia	2019–20	4,287	4.9	1870 (43.6)	398 (9.3)
6. Guinea	2018	3,869	4.4	1722 (44.5)	281 (7.3)
7. Liberia	2019–20	2,975	3.4	1282 (43.1)	792 (26.6)
8. Mali	2018	3,563	4.0	2275 (63.8)	438 (12.3)
9. Malawi	2015–16	8,615	9.7	2934 (34.1)	2265 (26.3)
10. Nigeria	2018	14,622	16.5	8343 (57.1)	1120 (7.7)
11. Rwanda	2019–20	5,108	5.8	690 (13.5)	1066 (20.9)
12. Sierra Leone	2019	5,587	6.3	2502 (44.8)	1522 (27.2)
13. Tanzania	2015–16	4,677	5.3	2055 (43.9)	867 (18.5)
14. Uganda	2016	6,596	7.5	2027 (30.7)	1345 (20.4)
15. South Africa	2018	2,868	3.2	972 (33.9)	618 (21.5)
16. Zimbabwe	2015	3,476	3.9	955 (27.5)	1310 (37.7)
**All countries**	**2015–2020**	**88,474**	**100.0**	**36274 (41.0)**	**14324 (16.2)**

### Variables

Anaemia was the outcome variable in our study. In the DHS, women of reproductive age were tested for anaemia through finger prick using the HemoCue hemoglobin testing system. The test was voluntary, and the results were made available to the women. Anaemia was measured using grams per deciliter (g/dl) and it was based on the level of haemoglobin concentration (Hb) in the blood [21]. Per the World Health Organization’s requirement [21], non-pregnant women with Hb less than 12.0g/dl were categorized as anaemic and coded as “1”. Non-pregnant women whose Hb level was 12.0g/dl and above were considered normal and was coded as “0” [22, 23].

Hormonal contraceptive use was the key explanatory variable in the study. The women were asked to indicate the contraceptive method they currently use. We dropped women who reported using an intrauterine device (IUD) and other modern methods due to a lack of accurate information as to the type of IUD and modern methods, respectively. Women who indicated using an oral contraceptive, injectable, and implants were said to use hormonal contraceptives, coded as “1 = yes”. The women who did not use any hormonal contraceptive were coded as “0 = n0” [22].

### Covariates

The covariates were included based on their association with anaemia from previous studies [22, 24–26]. Also, only the variables found in the DHS dataset across all the included countries were used. We grouped the variables into individual-level and contextual level. The individual-level covariates were women’s age, educational level, marital status, parity, cigarette smoking, health facility visit in the last 12 months, and body mass index. Place of residence, household wealth index, and geographic sub-regions were used as the contextual-level variables. The categories of the variables are shown in Table 2.

**Table 2:** Distribution of anaemia across the explanatory variables.

Variables	Weighted Frequency (n)	Weighted % Percentage (%)	Anaemia		
			No (%)	Yes (%)	P-value
**Hormonal contraceptive use**					<0.001
No	74,150	83.8	56.6	43.4	
Yes	14,324	16.2	71.3	28.7	
**Women’s age (years)**					<0.001
15–19	22,296	25.2	58.4	41.6	
20–24	14,725	16.6	60.4	39.6	
25–29	12,659	14.3	61.1	38.9	
30–34	11,124	12.6	59.7	40.3	
35–39	10,462	11.8	57.6	42.4	
40–44	8,810	10.0	56.9	43.1	
45–49	8,398	9.5	58.1	41.9	
**Level of education**					<0.001
No education	24,350	27.5	50.4	49.6	
Primary	27,177	30.7	63.8	36.2	
Secondary or higher	36,947	41.8	61.2	38.8	
**Marital status**					<0.001
Never in union	31,826	36.0	61.7	38.3	
Married	41,060	46.4	56.0	44.0	
Living with partner	7,238	8.2	62.2	37.8	
Widowed	2,856	3.2	58.3	41.7	
Divorced	2,218	2.5	62.1	37.9	
Separated	3,276	3.7	61.8	38.2	
**Parity**					<0.001
Zero birth	32,816	37.1	60.0	40.0	
One birth	10,173	11.5	59.6	40.4	
Two births	9,778	11.0	62.0	38.0	
Three births	8,828	10.0	60.0	40.0	
Four or more births	26,879	30.4	56.1	43.9	
**Body mass index**					<0.001
Thin	8,139	9.2	54.6	45.4	
Normal	55,172	62.4	58.0	42.0	
Overweight/obese	25,163	28.4	62.7	37.3	
**Smoke cigarettes**					0.405
No	87,698	99.1	59.0	41.0	
Yes	776	0.9	61.0	39.0	
**Health facility visit in the last 12 months**					<0.001
No	45,922	51.9	57.7	42.3	
Yes	42,552	48.1	60.4	39.6	
**Wealth index**					<0.001
Poorest	13,547	15.3	54.1	45.9	
Poorer	15,334	17.3	56.5	43.5	
Middle	16,832	19.0	57.8	42.2	
Richer	19,502	22.1	59.9	40.1	
Richest	23,259	26.3	63.6	36.4	
**Place of residence**					0.434
Urban	35,563	40.2	59.3	40.7	
Rural	52,911	59.8	58.8	41.2	
**Geographical sub-regions**					<0.001
Central	5,146	5.8	60.2	39.8	
Eastern	27,820	31.5	70.9	29.1	
Southern	14,959	16.9	67.5	32.5	
Western	40,549	45.8	47.6	52.4	

*P-values were generated from chi-square test

### Statistical analyses

We analyzed the data using Stata software version 17.0. We used percentages to summarize the prevalence of hormonal contraceptives and anaemia among women of reproductive age. Pearson chi-square test of independence was performed to determine the distribution of anaemia across hormonal contraceptive use and the covariates as well as examine the variables significant variables. We used multilevel binary logistic regression analysis to examine the association between hormonal contraceptives and anaemia, controlling for the covariates. Five (Model O-IV) were built to examine the association between anaemia and hormonal contraceptives. Model O was the empty model, and it denotes the variance in anaemia attributed to the primary sampling unit (PSU) with no explanatory variable or covariates. Model I was fitted to contain only hormonal contraceptives use. We included hormonal contraceptives and individual-level covariates in Model II. Model III contained hormonal contraceptives and the contextual level. Model IV was fitted to contain hormonal contraceptive use and all the covariates. We presented the results using crude odds ratio (cOR) and adjusted odds ratios (aOR), with their respective 95% confidence intervals (95% CIs). Statistical significance was set at p<0.05. Also, all five models were fitted to include fixed and random effect results. The random effects show the measure of variation in the anaemia based on the PSU (measured by Intra-Class Correlation [ICC]). On the other hand, the fixed effects denote the association between hormonal contraceptives and/or covariates and anaemia. Additionally, we tested for model fitness as well as model comparison using Akaike’s Information Criterion (AIC). Hence, the model with the least AIC value was chosen as the best-fitted model, which in our study is Model IV. We used the Stata command "mlogit" to execute the multilevel regression models. To account for disproportionate sampling and non-response, the "svyset" command was used, and weighting was done to account for the intricate nature of DHS data.

### Ethical consideration

Ethical clearance was not sought for our study since the dataset is freely available in the public domain. We sought permission for the Monitoring and Evaluation to Assess and Use Results Demographic and Health Surveys (MEASURE DHS and it was granted before using the dataset. The detailed information about the ethical issues regarding the DHS dataset can be freely accessed at http://goo.gl/ny8T6X.

## Results

### Prevalence of hormonal contraceptives and anaemia among the women in sub-Saharan Africa

Table 1 shows the prevalence of hormonal contraceptives use and anaemia among women in SSA. Overall, 16.2% of the women were using hormonal contraceptives and this ranged from 7.2% in Burundi to 37.7% in Zimbabwe. With the prevalence of anaemia, it was found that 41.0% of the women who participated in the study were anaemic, which also ranged from 13.5% in Rwanda to 58.0% in Benin.

### Distribution of anaemia across the explanatory variables

Table 2 presents the results of the distribution of anaemia across the explanatory variables. The results showed that 43.4% of women were not using hormonal contraceptives were anaemic whereas only 28.7% of hormonal contraceptive users were anaemic. With the covariates, anaemia was prevalent among women aged 40–44 (43.1%), those with no education (49.6%), those who were married (44.0%), those with four or more births (43.9%), those who were thin (45.4%), and those who had not visited a health facility visit in the last 12 months (42.3%). Also, the proportion of anaemia was high among women in the poorest wealth quintile (45.9%), those in rural areas (41.2%), and those in Western Africa (52.4%) (Table 2).

### Mixed-effect analysis of the association between hormonal contraceptive use and anaemia in sub-Saharan Africa

#### Fixed effects results

Table 3 shows the results of the mixed-effect analysis of the association between hormonal contraceptive use and anaemia in SSA. As shown in model IV, women who use hormonal contraceptives (aOR = 0.56; 95%CI = 0.53, 0.59) had lower odds of being anaemic compared to those who are not using hormonal contraceptives. With the covariates, women aged 45–49 (aOR = 0.71; 95%CI = 0.64, 0.79) had the lowest odds of being anaemic compared to those aged 15–19. With education, those in secondary or higher (aOR = 0.83; 95%CI = 0.78, 0.88) level of education had the lowest odds of being anaemic. Women in the richest wealth quintile (aOR = 0.81; 95%CI = 0.75, 0.87), those who were overweight/obese (aOR = 0.71; 95%CI = 0.66, 0.76), and those in Eastern Africa (aOR = 0.58; 95%CI = 0.53, 0.65) were less likely to be anaemic compared to those in the poorest wealth quintiles, those who were thin and those in central Africa respectively. However, women who were separated (aOR = 1.33; 95%CI = 1.18, 1.50), those with four or more births (aOR = 1.15; 95%CI = 1.05, 1.26), and those in Western Africa (aOR = 1.56; 95%CI = 1.41, 1.72) were more likely to be anaemic compared to those who were never in union, no history of birth, and those in Central Africa, respectively.

**Table 3:** Mixed-effect analysis of the association between hormonal contraceptive use and anaemia in sub-Saharan Africa.

Variables	Model O	Model I cOR [95% CI]	Model II aOR [95% CI]	Model III aOR [95% CI]	Model IV aOR [95% CI]
***Fixed effect results***					
**Hormonal contraceptive use**					
No		1.00	1.00	1.00	1.00
Yes		0.53*** [0.50, 0.56]	0.52*** [0.49, 0.55]	0.58*** [0.55, 0.61]	0.56*** [0.53, 0.59]
**Women’s age (years)**					
15–19			1.00		1.00
20–24			0.86*** [0.81, 0.92]		0.91** [0.85, 0.97]
25–29			0.73*** [0.68, 0.79]		0.80*** [0.74, 0.87]
30–34			0.71*** [0.64, 0.78]		0.81*** [0.74, 0.89]
35–39			0.71*** [0.65, 0.79]		0.83*** [0.75, 0.92]
40–44			0.68*** [0.62, 0.76]		0.83*** [0.75, 0.92]
45–49			0.58*** [0.53, 0.65]		0.71*** [0.64, 0.79]
**Level of education**					
No education			1.00		1.00
Primary			0.61*** [0.58, 0.64]		0.89*** [0.84, 0.94]
Secondary or higher			0.67*** [0.64, 0.71]		0.83*** [0.78, 0.88]
**Marital status**					
Never in union			1.00		1.00
Married			1.37*** [1.28, 1.47]		1.23*** [1.14, 1.31]
Living with partner			1.19*** [1.09, 1.30]		1.26*** [1.15, 1.38]
Widowed			1.17* [1.04, 1.33]		1.20** [1.06, 1.36]
Divorced			1.17* [1.02, 1.33]		1.23** [1.08, 1.40]
Separated			1.24*** [1.10, 1.39]		1.33*** [1.18, 1.50]
**Parity**					
Zero birth			1.00		1.00
One birth			1.13** [1.05, 1.22]		1.05 [0.97, 1.13]
Two births			1.06 [0.97, 1.15]		0.98 [0.90, 1.07]
Three births			1.17*** [1.07, 1.28]		1.07 [0.98, 1.17]
Four or more births			1.29*** [1.18, 1.41]		1.15** [1.05, 1.26]
**Body mass index**					
Thin			1.00		1.00
Normal			0.90** [0.84, 0.96]		0.90** [0.85, 0.97]
Overweight/obese			0.75*** [0.69, 0.81]		0.71*** [0.66, 0.76]
**Health facility visit in the last 12 months**					
No			1.00		1.00
Yes			0.99 [0.95, 1.03]		1.11*** [1.06, 1.15]
**Wealth index**					
Poorest				1.00	1.00
Poorer				0.90** [0.85, 0.96]	0.94* [0.88, 1.00]
Middle				0.84*** [0.79, 0.90]	0.91** [0.85, 0.97]
Richer				0.77*** [0.72, 0.82]	0.87*** [0.81, 0.94]
Richest				0.66*** [0.62, 0.71]	0.81*** [0.75, 0.87]
**Geographical sub-regions**					
Central				1.00	1.00
Eastern				0.63*** [0.57, 0.70]	0.58*** [0.53, 0.65]
Southern				0.73*** [0.66, 0.82]	0.73*** [0.65, 0.82]
Western				1.66*** [1.51, 1.84]	1.56*** [1.41, 1.72]
***Random effect model***					
PSU variance (95% CI)	0.470 [0.406, 0.545]	0.454 [0.391, 0.528]	0.504 [0.435, 0.584]	0.348 [0.295, 0.409]	0.366 [0.311, 0.430]
ICC	0.125	0.121	0.133	0.096	0.100
Wald chi-square	Reference	613.92 (<0.001)	1482.17 (<0.001)	2193.57 (<0.001)	2577.96 (<0.001)
**Model fitness**					
Log-likelihood	-165719.06	-164272.87	-161954.06	-158772.98	-157908.51
AIC	331442.1	328551.7	323954.1	317566	315877
*N*	88474	88474	88474	88474	88474
Number of clusters	1393	1393	1393	1393	1393

aOR = adjusted odds ratio; CI = Confidence Interval;

* *p* < 0.05,

** *p* < 0.01,

*** *p* < 0.001;

1.00 = Reference category; PSU = Primary Sampling Unit; ICC = Intra-Class Correlation; AIC = Akaike’s Information Criterion

#### Random effect results

The random effects results are shown in Table 3. Model 0 indicated that 13% of the variation in anaemia in SSA was attributed to intra-class correlation variation (ICC = 0.125). The variation between clusters decreased to 12% (0.121) in Model I but increased slightly to 13% again in Model II and decreased further to 0.096 in Model III and 0.100 in Mode IV. This further reiterates that the variations in the likelihood of anaemia in SSA are attributed to the clustering variation in PSUs. In addition, the last model, Model IV had the lowest AIC 315877, which shows that it was the best model to predict the association between hormonal contraceptive use and anaemia in SSA.

#### Association between hormonal contraceptive use and anaemia segregated by country

Table 4 shows the association between hormonal contraceptive use and anaemia disaggregated by country. It was found that in almost all the countries, women who use hormonal contraceptives were less likely to be anaemic compared to those who do not hormonal contraceptives with those in Malawi [aOR = 0.49;95%CI = 0.39, 0.62] and Liberia (aOR = 0.49; 95%CI = 0.37, 0.65) having the lowest odds.

**Table 4:** Association between hormonal contraceptive use and anaemia disaggregated by country.

Country	Model I cOR [95% CI]	Model II aOR [95% CI]
1. Benin	0.57*** [0.45, 0.73]	0.56*** [0.44, 0.71]
2. Burundi	0.66* [0.48, 0.91]	0.53*** [0.38, 0.74]
3. Cameroon	0.73* [0.57, 0.99]	0.81 [0.60, 1.10]
4. Ethiopia	0.87 [0.71, 1.07]	0.68** [0.55, 0.86]
5. Gambia	0.58*** [0.43, 0.77]	0.50*** [0.37, 0.68]
6. Guinea	1.14 [0.81, 1.61]	1.17 [0.82, 1.67]
7. Liberia	0.46*** [0.34, 0.61]	0.49*** [0.37, 0.65]
8. Mali	0.61** [0.44, 0.86]	0.64* [0.45, 0.90]
9. Malawi	0.49*** [0.40, 0.59]	0.49*** [0.39, 0.62]
10. Nigeria	0.69** [0.54, 0.87]	0.69** [0.54, 0.88]
11. Rwanda	0.57*** [0.42, 0.77]	0.58** [0.41, 0.81]
12. Sierra Leone	0.71*** [0.60, 0.83]	0.72*** [0.61, 0.86]
13. Tanzania	0.53*** [0.46, 0.61]	0.51*** [0.43, 0.59]
14. Uganda	0.48*** [0.36, 0.63]	0.50*** [0.37, 0.68]
15. South Africa	0.48*** [0.34, 0.69]	0.54** [0.38, 0.77]
16. Zimbabwe	0.54*** [0.48, 0.62]	0.52*** [0.44, 0.62]

## Discussion

Principally, the study tested the hypothesis that there is a statistically significant association between history of hormonal contraceptives use and the risk of anaemia among women in SSA. The findings support the hypothesis. Concerning the direction of the association, the study revealed that there is a significantly negative association. That is, women who had a history of hormonal contraceptives use were less likely to be anaemic compared to those who have no history of hormonal contraceptives use. This association remained significant after controlling for covariates. Thus, suggesting a probable protective effect of hormonal contraceptives use against iron deficiency and anaemia among women of reproductive age. Similar findings of a significantly negative association between history of hormonal contraceptives use and risk of anaemia have been reported in previous studies [7, 22, 23]. Plausibly, the observed findings could be explained from the point of reduction in menstrual bleeding associated with the use of hormonal contraceptives [27]. Available evidence suggests that women aged between 15–49 years lose 20–35 mL of blood on average per month during their menstrual flow; in some cases, this loss can reach 80 mL, resulting in an iron loss of more than 11 mg per month, hence, exacerbating the risk of anaemia [28]. However, the use of hormonal contraceptives such as combined oral contraceptive pills (COCP) can provide control of the menstrual cycle by thinning the endometrium (the lining of the womb that is shed during menstruation), thereby, reducing the risk of heavy bleeding, which serves as a protective factor against anaemia among women of reproductive age [29].

Less than half of women (41.0%) were anemic. This result is somewhat consistent with a previous study that found the prevalence of anaemia to be 42.2% [14]. Concerning the covariates, the study revealed that compared to adolescents, older women (i.e., 20 years and older) were less likely to be anemic. Similar findings have been reported in SSA [14] and Asia [30]. Unlike older women, adolescent girls may lack knowledge of proper dietary and nutrition, which are crucial for the prevention of anaemia [31]. Additionally, adolescents may not have the economic resources to afford the required nutritional needs, hence, placing them at higher risk of anaemia.

Consistent with previous studies [14, 32], the current study found lower risk of anaemia among women who had formal education compared with those with no formal education. Formal education has been linked to good knowledge on nutritional and dietary needs as well as better uptake of healthcare services [33, 34]. All of these could culminate in a significant reduction in the risk of anaemia among women of higher education. Relatedly, it was revealed that the risk of anaemia was significantly higher among women in union compared to those who had never married. The result aligns with previous studies that have found a similar association between marital status and the risk of anaemia [14, 24]. This might be because married women frequently become pregnant, which increases their risk of hemorrhage before, during, and after delivery and puts them at a higher risk of anaemia [35].

The results from this indicates that the risk of anaemia reduces with higher wealth index. Similar findings have been reported in SSA [14] and Asia [30]. It is possible that women in poorer households find it more difficult to afford healthy food [36], pay for health care [37], and maintain decent hygiene [38], which could have contributed to the high level of anaemia. Moreover, women from poor wealth status are less likely to have autonomy in making household decisions, which may affect the quality of their dietary decisions. Hence, increasing their risk of anaemia.

Also, the study revealed multiparous women have higher risk of anaemia compared to nulliparous women. This finding is in line with a related study from SSA [14] and the Lao People’s Democratic Republic [39]. Similarly, there was significant geographical differences in the risk of anaemia with women from Western Africa having a higher likelihood of being anaemic. It is uncertain the reasons for the findings. However, this could be due to cultural differences that influence the dietary and nutrition decisions of women in Western African countries compared with other SSA countries. Nevertheless, the present study does not provide any concrete evidence on what may be accounting for the geographic differences in the risk of anaemia among women in the reproductive age. Therefore, further studies are recommended to ascertain the geographical differences in the association between sub-regions in SSA.

### Strengths and limitations

The strength of this study lies in the use of a large, nationally representative dataset from sub-Saharan African countries where anaemia is considered a serious public health concern. Also, the study followed appropriate methodologies to analyze the data. Nonetheless, some limitations must be considered. As the study was based on the DHS data that used cross-sectional design, it is difficult to establish causal inference between hormonal contraceptives use and the risk of anaemia. Given that the use of hormonal contraceptives was based on self-reported data, there is the possibility of social desirability and recall bias.

## Conclusion

Our study has shown that there is a significant association between hormonal contraceptives use and the risk of anaemia among women of reproductive age. The study underscores the importance of promoting the use of hormonal contraceptives in communities and regions that have a high burden of anaemia among women. Specifically, health promotion interventions aimed at promoting the use of hormonal contraceptives among women must be tailored to meet the needs of adolescents, multiparous women, those in the poorest wealth index, and women in union as these sub-populations were at significantly higher risk of anaemia in SSA.

## Supporting information

S1 File (DOCX)
