# Supplementary material for: Relationship between history of hormonal contraceptive use and anaemia status among women in sub-Saharan Africa: A large population-based study
Source: PLoS One. 2023 Jun 14;18(6):e0286392. doi: 10.1371/journal.pone.0286392 (PMC10266693; doi:10.1371/journal.pone.0286392)
Supplement: S1 File — (DOCX) [file pone.0286392.s001.docx]

Supporting Information file 1.

| **Country** | **Source of data** |
| --- | --- |
| Benin | <https://dhsprogram.com/data/dataset/Benin_Standard-DHS_2017.cfm?flag=1> |
| Burundi | <https://dhsprogram.com/data/dataset/Burundi_Standard-DHS_2016.cfm?flag=1> |
| Cameroon | <https://dhsprogram.com/data/dataset/Cameroon_Standard-DHS_2018.cfm?flag=1> |
| Ethiopia | <https://dhsprogram.com/data/dataset/Ethiopia_Standard-DHS_2016.cfm?flag=1> |
| Gambia | <https://dhsprogram.com/data/dataset/Gambia_Standard-DHS_2019.cfm?flag=1> |
| Guinea | <https://dhsprogram.com/data/dataset/Guinea_Standard-DHS_2018.cfm?flag=1> |
| Liberia | <https://dhsprogram.com/data/dataset/Liberia_Standard-DHS_2019.cfm?flag=1> |
| Mali | <https://dhsprogram.com/data/dataset/Mali_Standard-DHS_2018.cfm?flag=1> |
| Malawi | <https://dhsprogram.com/data/dataset/Malawi_Standard-DHS_2015.cfm?flag=1> |
| Nigeria | <https://dhsprogram.com/data/dataset/Nigeria_Standard-DHS_2018.cfm?flag=1> |
| Rwanda | <https://dhsprogram.com/data/dataset/Rwanda_Standard-DHS_2019.cfm?flag=1> |
| Sierra Leone | <https://dhsprogram.com/data/dataset/Sierra-Leone_Standard-DHS_2019.cfm?flag=1> |
| Tanzania | <https://dhsprogram.com/data/dataset/Tanzania_Standard-DHS_2015.cfm?flag=1> |
| Uganda | <https://dhsprogram.com/data/dataset/Uganda_Standard-DHS_2016.cfm?flag=1> |
| South Africa | <https://dhsprogram.com/data/dataset/South-Africa_Standard-DHS_2016.cfm?flag=1> |
| Zimbabwe | <https://dhsprogram.com/data/dataset/Zimbabwe_Standard-DHS_2015.cfm?flag=1> |
